# Effect of sodium zirconium cyclosilicate on hyperkalemia after parathyroidectomy in secondary hyperparathyroidism patients with maintenance hemodialysis: A randomized trial

**DOI:** 10.1097/MD.0000000000040917

**Published:** 2024-12-27

**Authors:** Jing Kang, Sijia Li, Jinglin Su, Zhixue Xiao, Siyi Zhang, Shuangxin Liu, Pingjiang Ge

**Affiliations:** a Department of Otolaryngology, Guangdong Provincial People’s Hospital (Guangdong Academy of Medical Sciences), Southern Medical University, Guangzhou City, P.R. China; b Department of Nephrology, Guangdong Provincial People’s Hospital (Guangdong Academy of Medical Sciences), Southern Medical University, Guangzhou City, P.R. China; c Department of Otolaryngology, School of Medicine South China University of Technology, Guangzhou City, P.R. China.

**Keywords:** hemodialysis, hyperkalemia, hyperparathyroidism, parathyroidectomy, sodium zirconium cyclosilicate

## Abstract

**Background:**

Postoperative hyperkalemia is 1 common complication after parathyroidectomy (PTX), which requires close monitoring and prompt treatment. This study aimed to determine whether using sodium zirconium cyclosilicate (SZC) would lower the risk of hyperkalemia in patients with maintenance hemodialysis after PTX.

**Methods:**

Sixty-two patients with secondary hyperparathyroidism (SHPT) were randomly divided into the experimental and control groups. Patients in the experimental group were required to take 10 g of SZC before PTX. Laboratory chemistries were obtained before and after surgery.

**Results:**

Parathyroid hormone (PTH) decreased dramatically in the experimental and control groups after PTX. There were no significant differences in serum potassium ion (K^+^) between the 2 groups at 6 am on the day of surgery and immediately after surgery. However, serum potassium in the experimental group at 9 pm on the day of surgery was significantly lower than in the control group. Three patients with severe hyperkalemia in the control group received emergency hemodialysis or insulin and glucose treatment, while none in the experimental group required hemodialysis. Serum calcium levels declined immediately after the operation, but no significant differences were found between these 2 groups at all time points.

**Conclusion:**

SZC has the potential to reduce the occurrence of hyperkalemia and avoid urgent hemodialysis after PTX. We recommended that SZC could be used routinely in SHPT patients on the day of PTX surgery.

## 1. Introduction

Secondary hyperparathyroidism (SHPT) is 1 kind of severe manifestation of chronic kidney disease, especially in end-stage renal disease (ESRD) patients. SHPT is characterized by inappropriate synthesis and secretion of PTH accompanied by parathyroid cell hyperplasia.^[1]^ Persistence of PTH secretion results in metabolic disorders of calcium (Ca), phosphate(P), and calcitriol, which play a vital role in developing bone disease and vascular calcification.^[2]^ Elevated PTH level is an important trigger for fracture, hyperphosphatemia, anemia, and cardiovascular calcification that worsens the health-related quality of life and increases mortality.^[1,3,4]^

Although medical treatment, including phosphate binders, vitamin D and its analogs, and calcimimetics, improves target levels for all the metabolic abnormalities associated with SHPT, a significant proportion of refractory SHPT patients have inadequately controlled serum PTH, P, and Ca levels.^[5]^ Furthermore, the Eastern European countries, especially the developing area, showed a poorer control of the biochemical parameters (Ca, P and PTH) than Western European countries, demonstrating that economic level impacted the management of ESRD patients.^[6]^ A possible explanation for these results may be inadequate hemodialysis and medical control. The financial burden of ESRD patients would impose restrictions on medical treatment. Therefore, Parathyroidectomy (PTX) has been regarded as the first-line treatment for severe SHPT.^[7]^ To control secondary hyperparathyroidism, approximately 29% of patients with ESRD undergo parathyroidectomy.^[8]^ Successful PTX may promptly reduce serum PTH levels and alleviate clinical symptoms.^[9]^

However, significant concern about the risk associated with PTX remained answered. Postoperative hyperkalemia is 1 common complication after PTX. With an incidence of 25% to 80%, in previous research, hyperkalemia has been reported during and immediately after PTX, leading to serious consequences.^[8,10–12]^ Therefore, extra care must be taken for postoperative hyperkalemia. PTX patients should perform emergency treatments immediately after severe hyperkalemia confirmation to avoid accelerated repolarization, reduced conduction velocity, and even precipitate fatal arrhythmias.^[13]^ However, several risks should be considered when hemodialysis during post-anesthesia care. Hemodialysis without anticoagulation may lead to extracorporeal circuit clotting. Additionally, immediate hemodialysis after surgery could result in hypoglycemia due to prolonged fasting and catheter disconnection due to an unconscious condition.

Sodium zirconium cyclosilicate (SZC, Lokelma^TM^) is a non-absorbed, non-polymer zirconium silicate compound that preferentially entraps ammonium and potassium ions and exchanges them for sodium and hydrogen ions in the gastrointestinal tract, thereby increasing fecal potassium excretion and decreasing serum potassium levels during the 48 hours of treatment.^[14]^ Results from multinational, phase III research have confirmed the serum potassium-lowering efficacy and safety profile of SZC in adults with hyperkalemia.^[15,16]^ Nevertheless, the effects of SZC during the perioperative period remained unknown. This study aimed to determine whether using SZC would lower the risk of hyperkalemia in ESRD patients after PTX and prevent emergency dialysis following surgery.

## 2. Materials and methods

### 2.1. Trial design

This study was a single-blind, randomized clinical trial. All patients signed the informed consent form before being randomized into our experiment. After screening, participants were divided into 2 groups (control and experimental groups). Block randomization was performed using a computer-generated random number list the principal investigator prepared. The principal investigator will enroll and assign participants to interventions. This prospective comparative study was approved by the Research Ethics Committee of Guangdong Provincial People’s Hospital (protocol no. GDREC2019512H) and registered with clinicaltrials.gov (protocol NCT05382988).

### 2.2. Subjects

The sample size was determined based on previous studies. Kosiborod et al^[16]^ evaluated the efficacy and safety of SZC in patients with hyperkalemia. A significant change in potassium (−0.5 mEq/L; 95% CI, −0.6 to − 0.5) was noted 4 hours after the 10 g dose compared with baseline (*P* < .001). The test level a was set to 0.05, and the test efficacy (1-β) was set to 0.80, and the minimum sample size of n = 50 was calculated with an allocation ratio of 1:1.

The inclusion criteria are as follows: 1. Patients diagnosed with SHPT 2. Patients exhibiting hyperparathyroid hormone levels exceeding 800 pg/mL, with or without hypercalcemia, and presenting definitive associated symptoms such as bone pain, skin pruritus, and fractures. 3. Absence of evident contraindications arising from cardiopulmonary and systemic conditions for tolerating general anesthesia surgery. 4. Absence of known allergy to SZC. The exclusion criteria are as follows: 1. Individuals aged over 70 years; 2. Those with significant cardiopulmonary dysfunction, mental health conditions, or other specific circumstances that would preclude the safe administration of general anesthesia.

A total of sixty-two ESRD MHD with SHPT patients were recruited into this study between November 2016 and December 2021 from the Otolaryngology Head and Neck Surgery department of Guangdong Provincial People’s Hospital. All patients were randomly and equally divided into the experimental and control groups. The related population information is provided in Table 1.

**Table 1:** Demographics and clinical characteristics of the study population.

Characteristic	Experimental group	Control group
Number	31	31
Age (yr)	47.903 ± 12.413	51.484 ± 11.316
Male: female	16:15	14:17
Dialysis duration (yr)	8.336 ± 3.612	6.984 ± 2.722
Creatinine (µmol/L)	916.872 ± 223.425	952.448 ± 173.171
Alkaline phosphatase (U/L)	568.226 ± 491.521	375.516 ± 320.187
Preoperative serum PTH (pg/mL)	2182.797 ± 1119.044	1822.890 ± 714.008
Postoperative serum PTH (pg/mL)	57.934 ± 49.569	78.208 ± 73.464

### 2.3. Clinical procedure

The experimental group was required to take SZC 10 g immediately after the blood test at 6 am on the surgery day, while no additional medicine was taken in the control group. All patients were treated with PTX by 1 experienced surgeon in the Otolaryngology Head and Neck Surgery department in Guangdong Provincial People’s Hospital, who had dialysis within 24 hours before PTX. All the operations were performed around noon to avoid the risk of aspiration during general anesthesia after taking the SZC. The surgical procedure was as follows. A low collar incision 6 to 7 cm in length was operated. All inferior and superior parathyroids were removed. A fragment of parathyroid tissue (60–100 mg) is placed into the brachioradialis muscle’s surface for auto-transplantation. Several factors were not applied during surgery and anesthesia, such as succinylcholine, calcium, and blood product contributing to hyperkalemia or hypokalemia. The calcium supplements were administered intravenously in all patients after PTX to prevent postoperative hypocalcemia. Based on our previous research,^[17]^ the amount of postoperative calcium supplement every 6 hours was calculated according to the equations.


$$\begin{array}{l} Y=\left(0.249X_{1}-0.035X_{2}+18.406\right)/11 \end{array}$$

Where *Y* = 10% gluconate calcium (mL) intravenous, *X*_1_ = preoperative PTH level (pg/mL), *X*_2_ = preoperative alkaline phosphatase level (U/L).

### 2.4. Laboratory chemistry measurement

Alkaline phosphatase and creatinine were measured before surgery. Serum PTH was checked 24 hours before surgery and on the first day after surgery. Serum potassium levels and serum calcium levels were checked at 3 time points, which were t0 (6 am on the day of surgery), t1 (immediately after surgery), and t2 (9 pm on the day of surgery), respectively.

### 2.5. Statistical analysis

Statistical analysis of the data was carried out using SigmaPlot14 (Systat, San Jose, CA). Normality was evaluated using the Shapiro–Wilk test, and tests for an equal variance were also performed. The data were compared at the different time points in 2 groups using a 2-way analysis of variance (ANOVA) with repeated measures. The between-subjects effect (main effect of the group), the within-subject effect (main effect of the time), and the interaction between the group and the time were tested. When the ANOVA showed significant differences (*P* < .05), a TUKEY post hoc test was computed on the pairwise comparisons. The significance level was established at 0.05, with a 95% confidence interval.

## 3. Results

### 3.1. Serum PTH

**Figure 1.**
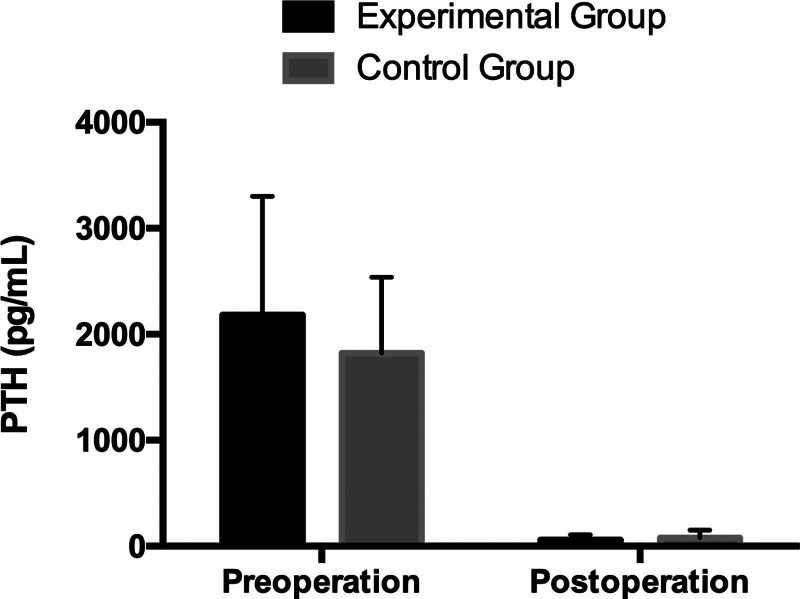
Variation of PTH in the experimental group and the control group.

The main effect of time in PTH was significant (*P* < .001). There has been a sharp drop in PTH after PTX in both experimental and control groups, shown in Table 1 and Figure 1. However, no significant effects were detected in the main effect of the group and the interaction (*P* = .154, *P* = .106).

### 3.2. Serum potassium

**Figure 2.**
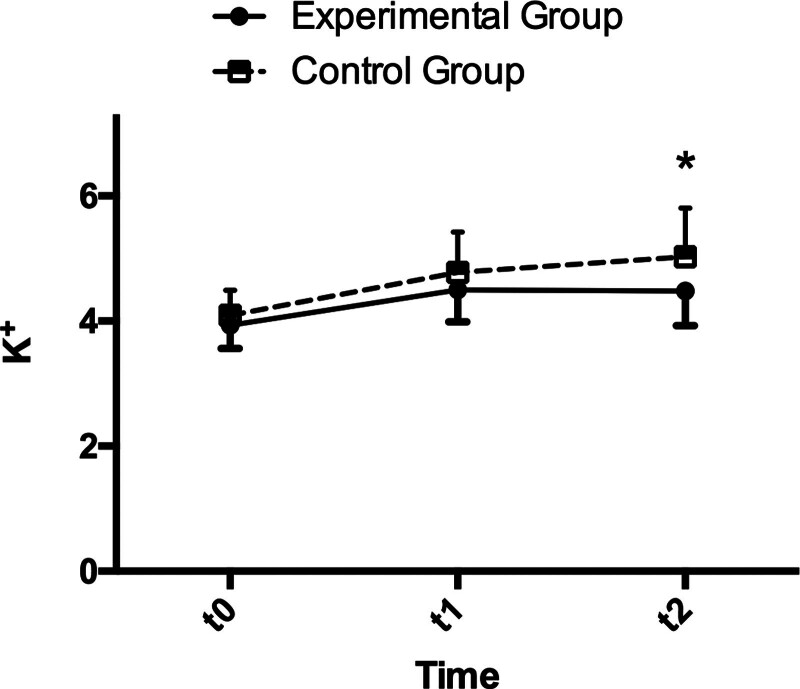
Variation of K^+^ in the experimental group and the control group at 3 time points. (* *P* < .05).

The main effect of group, time, and interaction between group and time in K^+^ were significant, as shown in Table 2 and Figure 2 (*P* = .011, *P* < .001, *P* = .018, respectively). *t*-Tests for time effects revealed substantial increases in both groups between t1, t2 compared with t0 (*P* < .001). No significant change was found in the experimental group between t1 and t2 (d = 0.008, *P* = .995), while a considerable rise was obtained in the control group (d = 0.250, *P* = .017, Table 3). Between-group comparisons of K^+^ detected a significant difference at t2 between the experimental and control group, whereas no significant differences were found in t0 and t1 between these 2 groups (Table 4).

**Table 2:** Results from 2-way ANOVA with repeated measures for K^+^.

Source of variation	K^+^	
	*F*	*P*
Group	7.316	.011*
Time	85.181	<.001*
Group × Time	4.287	.018*

* Comparisons with significant results.

**Table 3:** Within-group comparisons of K^+^ between different time points.

Dependent variables	Time	Experimental group		Control group	
		Diff of means	*P*	Diff of means	*P*
K^+^ (mmol/L)	t0 vs t1	0.568	<.001*	0.683	<.001*
	t0 vs t2	0.559	<.001*	0.993	<.001*
	t1 vs t2	0.008	.995	0.250	.017*

* Comparisons with significant results.

**Table 4:** Between-group comparisons of K^+^ between 2 groups.

Dependent variables	Time	Experimental group	Control group	*P*
K^+^ (mmol/L)	t0	3.932 ± 0.371	4.093 ± 0.401	.261
	t1	4.499 ± 0.512	4.776 ± 0.648	.056
	t2	4.491 ± 0.556	5.025 ± 0.779	<.001*

* Comparisons with significant results.

In control group, the serum potassium level increased from a mean of 4.09 mmol/L preoperatively to 5.03 mmol/L at t2. There were 19.4% (6/31) patients whose potassium levels rose above the normal limit (5.30 mmol/L in our hospital) and 3.2% (1/31) patients above the critical limit (6.00 mmol/L) at t1, while 32.3% (10/31) patients whose potassium levels were found to rise above the normal limit and 9.7% (3/31) patients were above the critical limit at t2. Of the 3 patients with severe hyperkalemia, 2 received emergency hemodialysis, and the other was treated with insulin and glucose (40 mL 50% glucose + 5U insulin) intravenous injection after t2.

While in the experimental group, the serum potassium level increased from a mean of 3.932 mmol/L preoperatively to 4.491 mmol/L at t2. A significantly lower potassium level was obtained in the experimental group compared with the control group at t2. Two patients whose potassium levels rose above the normal limit at t1, but fell back to normal at t2, while only 1 patient whose potassium level was found to rise above the normal limit at t2. No patient was above the critical limit at t1 and t2. No emergency hemodialysis and other treatment were performed on experimental group.

### 3.3. Serum Ca^2+^

**Figure 3.**
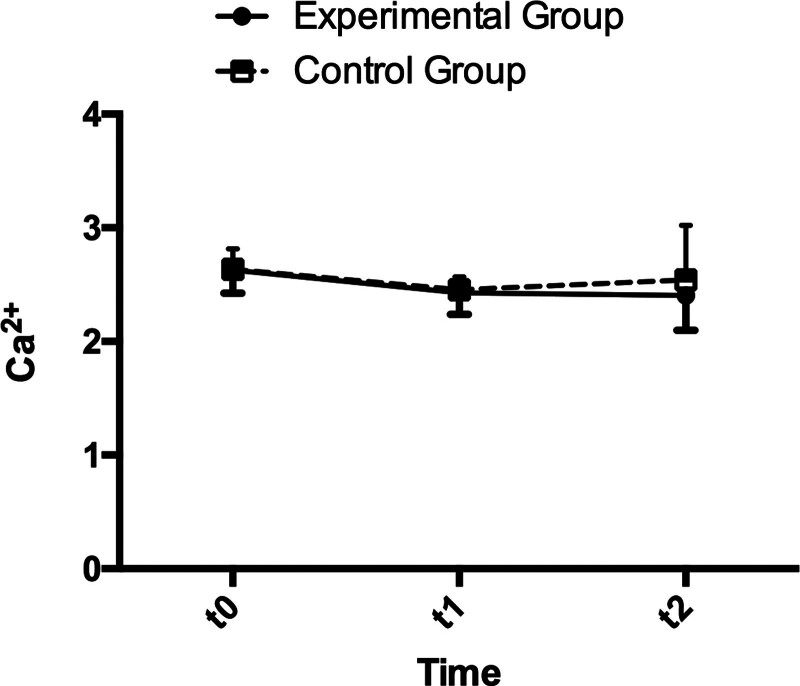
Variation of Ca^2+^ in the experimental group and the control group at 3 time points.

The main effect of time was found significant in Ca^2+^, as shown in Table 5 (*P* < .001). Figure 3 revealed that there had been a considerable decline in Ca^2+^ between t1 compared with t0 in both groups (d = 0.198, *P* = .001; d = 0.183, *P* = .004 respectively, Table 6), but no significant difference was found between these 2 groups in all 3 time points (Table 7).

**Table 5:** Results from 2-way ANOVA with repeated measures for Ca^2+^.

Source of variation	Ca^2+^	
	*F*	*P*
Group	1.096	.303
Time	15.890	<.001*
Group × time	1.408	.252

* Comparisons with significant results are denoted.

**Table 6:** Within-group comparisons of Ca^2+^ between different time points.

Dependent variables	Time	Experimental group		Control group	
		Diff of means	*P*	Diff of means	*P*
Ca^2+^ (mmol/L)	t0 vs t1	0.198	.001*	0.183	.004*
	t0 vs t2	0.222	<.001*	0.094	.209
	t1 vs t2	0.024	.9052	0.089	.244

* Comparisons with significant results.

**Table 7:** Between-group comparisons of Ca^2+^ between 2 groups.

Dependent variables	Time	Experimental group	Control group	*P*
Ca^2+^ (mmol/L)	t0	2.627 ± 0.201	2.636 ± 0.179	.898
	t1	2.429 ± 0.191	2.454 ± 0.113	.734
	t2	2.405 ± 0.308	2.543 ± 0.480	.063

*Comparisons with significant results.

## 4. Discussion

Prior studies have noted the critical association of PTX with improved survival rates and cardiovascular outcomes. Kestenbaum et al^[18]^ retrospectively analyzed 4558 patients and found a significant reduction in long-term mortality after PTX surgery. A similar result has also been detected in another study from Taiwan.^[19]^ Additionally, in Komaba et al study^[20]^ PTX was significantly associated with lower death rates from heart failure and cardiac arrest. Although a long-term follow-up has not been conducted in our study, PTH decreased back to normal immediately after surgery. Moreover, serum calcium level also declined immediately after the operation. The results confirmed that PTX was an efficient and effective method to improve metabolic abnormalities.

For now, no medical approach is likely to entirely prevent the need for PTX. However, the remaining questions should be investigated to avoid the risks associated with PTX.^[21]^ The probability of hyperkalemia during the perioperative period cannot be ignored. In Li et al study,^[10]^ the potassium levels immediately rose above the normal limit (5.30 mmol/L in the author’s hospital) in 28 of the 108 patients (25.9%) and above the critical limit (6.00 mmol/L) in 16 of the 108 patients (14.8%) after PTX. The exact causes of elevated serum potassium levels are unclear, but the tissue destruction resulting from surgery and the efflux of potassium from the intracellular compartment may be plausible mechanisms.^[11,22]^ A decreased calcium ions in an extracellular fluid resulting from the withdrawal of serum PTH after PTX can promote the influx of sodium ions into skeletal muscle cells under the mechanism of membrane barrier action of the sodium-calcium exchanger.^[14]^ This tendency counteracts sodium ion efflux and potassium ion influx powered by the activation of the Na/K ATPase pump, thereby explaining the increased serum potassium level during and after PTX.^[11,23,24]^

Therefore, hemodialysis may be required immediately after surgery or in the operating theater for persistent intraoperative hyperkalemia in chronic kidney patients.^[8]^ However, several risks should be considered when hemodialysis during post-anesthesia care. Systemic anticoagulation, such as unfractionated heparin (UFH), is essential for hemodialysis to avoid extracorporeal circuit clotting, stemming from endothelial damage and perturbations in the metabolism, expression, and activity of certain procoagulant factors. Paradoxically, the potential risk of bleeding rises in hemodialysis patients using anticoagulation. The incidence rate of major bleeding events was estimated to be 3.1 to 10.8 per 100 person-years in hemodialysis patients.^[25,26]^ The increased tendency to bleed would lead to devastating complications after PTX, such as wound uncontrollable bleeding and dyspnea. Although immediate hemodialysis after PTX can be done with heparin-free regional anticoagulation, this approach often results in circuit blockages, leading to insufficient hemodialysis time and reduced treatment effectiveness. In addition, immediate hemodialysis after surgery may result in hypoglycemia due to prolonged fasting and catheter disconnection due to an unconscious condition. Emergency hemodialysis after PTX is risky and challenging; thus, this study aimed to find a therapy to control potassium levels to avoid emergency hemodialysis after PTX.

SZC increases fecal potassium excretion and lowers serum potassium levels by binding potassium ions, demonstrated to reduce serum potassium to normal levels within 48 hours in hyperkalemia patients.^[16,27]^ In 1 multicenter trial, a significant (*P* < .005) reduction was seen from baseline as early as 1 hour after taking the SZC, with substantial (*P* < .005) reductions apparent at all subsequent time points (2, 4, 24 hours) during the 48 hours.^[16]^ In general, the recommended dosage of SZC is 10 g 3 times daily for patients with hyperkalemia for the correction period and 10 g once daily for the maintenance period,^[14]^ so we chose SZC 10 g once as our intervention dosage. Furthermore, SZC was found to have the most pronounced effect within the initial 4 hours. Therefore, we recommend administering SZC on the morning of the surgery to ensure optimal potassium reduction during the perioperative period.^[16]^

Similar to other studies,^[28,29]^ an upward trend has been found in serum potassium after general anesthesia surgery in our control group. Three patients’ potassium levels were above the critical limit at t2. The rate of patients with hyperkalemia further confirmed the cardiovascular risk after PTX and the importance of close monitoring, and prompt treatment. The experimental group found significantly lower serum potassium level than the control group. Furthermore, no patient was found above the critical limit in the potassium lever after surgery in the experimental group. The results displayed that SZC had the significant effects of suppressing the gradual rise in potassium level, which offer the possibility of avoiding dialysis on the day of surgery and guaranteeing safety during the perioperative period.

The safety profile of SZC across various studies has been confirmed. SZC is generally well tolerated during the maintenance period of 12 months in adults with hyperkalemia.^[16]^ The most commonly reported adverse reactions across the SZC dosages were hypokalemia, constipation, and edema-related events.^[15]^ These adverse reactions were primarily reported in the maintenance period and were thought to be dose-dependent.^[14]^ No noticeable side effects, including hypokalemia, edema, and digestive complications, were found in our study with the single dose of SZC 10 g. Additionally, as SZC is available as a powder for oral suspension, the patients were required to take SZC 4 hours before general anesthesia to avoid the risk of aspiration. No anesthesia complication during the operation occurred in our study.

The study’s limitation pertained to its single-blinded nature, where only the patients were unaware of their group assignment (experimental or control). Furthermore, the control group was not administered a placebo. However, given that the results relied on objective metrics such as serum potassium and PTH, it was indicated that this experimental design would not significantly impact the results and outcome.

Overall, this study strengthened the idea that SZC is a safe and remarkable therapy for preventing and managing hyperkalemia during PTX, which can avoid emergency dialysis on the day of surgery and lower the occurrence of hyperkalemia. We recommended that SZC could be used routinely in SHPT patients on the day of PTX surgery.

## Author contributions

**Conceptualization:** Sijia Li, Shuangxin Liu.

**Funding acquisition:** Shuangxin Liu, Pingjiang Ge.

**Investigation:** Jing Kang, Sijia Li, Siyi Zhang.

**Methodology:** Jing Kang, Jinglin Su, Zhixue Xiao, Pingjiang Ge.

**Software:** Jinglin Su.

**Supervision:** Siyi Zhang, Pingjiang Ge.

**Validation:** Sijia Li, Pingjiang Ge.

**Writing – original draft:** Jing Kang.

**Writing – review & editing:** Shuangxin Liu, Pingjiang Ge, Jing Kang.

## References

[1] Mizobuchi M Ogata H Koiwa F. Secondary hyperparathyroidism: pathogenesis and latest treatment. Ther Apher Dial. 2019;23:309–18. 30411503 10.1111/1744-9987.12772

[2] Rodríguez-Ortiz ME Rodríguez M. Recent advances in understanding and managing secondary hyperparathyroidism in chronic kidney disease. F1000Res. 2020;9:F1000 Faculty Rev–1077. doi: 10.12688/f1000research.22636.1.

[3] Kalantar-Zadeh K Kuwae N Regidor DL. Survival predictability of time-varying indicators of bone disease in maintenance hemodialysis patients. Kidney Int. 2006;70:771–80. 16820797 10.1038/sj.ki.5001514

[4] Young EW Albert JM Satayathum S. Predictors and consequences of altered mineral metabolism: the dialysis outcomes and practice patterns study. Kidney Int. 2005;67:1179–87. 15698460 10.1111/j.1523-1755.2005.00185.x

[5] Fernández-Martín JL Martínez-Camblor P Dionisi MP; COSMOS group. Improvement of mineral and bone metabolism markers is associated with better survival in haemodialysis patients: the COSMOS study. Nephrol Dial Transplant. 2015;30:1542–51. 25920921 10.1093/ndt/gfv099

[6] Fernández-Martín JL Carrero JJ Benedik M. COSMOS: the dialysis scenario of CKD-MBD in Europe. Nephrol Dial Transplant. 2013;28:1922–35. 23166310 10.1093/ndt/gfs418

[7] Narayan R Perkins RM Berbano EP. Parathyroidectomy versus cinacalcet hydrochloride-based medical therapy in the management of hyperparathyroidism in ESRD: a cost utility analysis. Am J Kidney Dis. 2007;49:801–13. 17533023 10.1053/j.ajkd.2007.03.009

[8] Bajaj Y Roberts S Simon D Snowden C Gianopoulos I England RJ. Intra-operative hyperkalemia: a serious but under recognised complication of renal parathyroidectomy - a prospective study: how we do it. Clin Otolaryngol. 2011;36:69–72. 21414155 10.1111/j.1749-4486.2011.02252.x

[9] Evenepoel P Claes K Kuypers D Maes B Vanrenterghem Y. Impact of parathyroidectomy on renal graft function, blood pressure and serum lipids in kidney transplant recipients: a single centre study. Nephrol Dial Transplant. 2005;20:1714–20. 15919696 10.1093/ndt/gfh892

[10] Li S Liu S Chen Q. Clinical predictor of postoperative hyperkalemia after parathyroidectomy in patients with hemodialysis. Int J Surg. 2018;53:1–4. 29548699 10.1016/j.ijsu.2018.03.003

[11] Yang G Wang J Sun J Zha X Wang N Xing C. Perioperative hyperkalemia in hemodialysis patients undergoing parathyroidectomy for renal hyperparathyroidism. Intern Emerg Med. 2019;14:1065–71. 30648222 10.1007/s11739-019-02031-5

[12] Yang YL Lu HF Chung KC Jawan B Chou FF. Young age, male sex, and end-stage renal disease with secondary hyperparathyroidism as risk factors for intraoperative hyperkalemia during parathyroidectomy. J Clin Anesth. 2015;27:195–200. 25434503 10.1016/j.jclinane.2014.06.015

[13] Putcha N Allon M. Management of hyperkalemia in dialysis patients. Semin Dial. 2007;20:431–9. 17897250 10.1111/j.1525-139X.2007.00312.x

[14] Hoy SM. Sodium zirconium cyclosilicate: a review in hyperkalaemia. Drugs. 2018;78:1605–13. 30306338 10.1007/s40265-018-0991-6 PMC6433811

[15] Packham DK Rasmussen HS Lavin PT. Sodium zirconium cyclosilicate in hyperkalemia. N Engl J Med. 2015;372:222–31. 25415807 10.1056/NEJMoa1411487

[16] Kosiborod M Rasmussen HS Lavin P. Effect of sodium zirconium cyclosilicate on potassium lowering for 28 days among outpatients with hyperkalemia: the HARMONIZE randomized clinical trial. JAMA. 2014;312:2223–33. 25402495 10.1001/jama.2014.15688

[17] Ge P Liu S Sheng X. Serum parathyroid hormone and alkaline phosphatase as predictors of calcium requirements after total parathyroidectomy for hypocalcemia in secondary hyperparathyroidism. Head Neck. 2018;40:324–9. 28963816 10.1002/hed.24965

[18] Kestenbaum B Andress DL Schwartz SM. Survival following parathyroidectomy among United States dialysis patients. Kidney Int. 2004;66:2010–6. 15496173 10.1111/j.1523-1755.2004.00972.x

[19] Ho LC Hung SY Wang HH; Tainan RENal Disease Study (TRENDS) group. Parathyroidectomy associates with reduced mortality in Taiwanese dialysis patients with hyperparathyroidism: evidence for the controversy of current guidelines. Sci Rep. 2016;6:19150. 26758515 10.1038/srep19150 PMC4725823

[20] Komaba H Taniguchi M Wada A Iseki K Tsubakihara Y Fukagawa M. Parathyroidectomy and survival among Japanese hemodialysis patients with secondary hyperparathyroidism. Kidney Int. 2015;88:350–9. 25786097 10.1038/ki.2015.72

[21] Eidman KE Wetmore JB. The role of parathyroidectomy in the management of secondary hyperparathyroidism. Curr Opin Nephrol Hypertens. 2017;26:516–22. 28985191 10.1097/MNH.0000000000000365

[22] Zou Y Zhang L Zhou H Yang Y Yang M Di J. Risk factors of hyperkalemia after total parathyroidectomy in patients with secondary hyperparathyroidism. Ren Fail. 2020;42:1029–31. 33028124 10.1080/0886022X.2020.1803088 PMC7580569

[23] Gonzalez-Serratos H Hilgemann DW Rozycka M Gauthier A Rasgado-Flores H. Na-Ca exchange studies in sarcolemmal skeletal muscle. Ann N Y Acad Sci. 1996;779:556–60. 8659879 10.1111/j.1749-6632.1996.tb44837.x

[24] Nielsen OB Harrison AP. The regulation of the Na+, K+ pump in contracting skeletal muscle. Acta Physiol Scand. 1998;162:191–200. 9578365 10.1046/j.1365-201X.1998.00297.x

[25] Holden RM Harman GJ Wang M Holland D Day AG. Major bleeding in hemodialysis patients. Clin J Am Soc Nephrol. 2008;3:105–10. 18003768 10.2215/CJN.01810407 PMC2390984

[26] Phelan PJ O’Kelly P Holian J. Warfarin use in hemodialysis patients: what is the risk? Clin Nephrol. 2011;75:204–11. 21329630 10.5414/cn106481

[27] Spinowitz BS Fishbane S Pergola PE; ZS-005 Study Investigators. Sodium zirconium cyclosilicate among individuals with hyperkalemia: a 12-month phase 3 study. Clin J Am Soc Nephrol. 2019;14:798–809. 31110051 10.2215/CJN.12651018 PMC6556727

[28] Ciriaco P Casiraghi M Melloni G. Pulmonary resection for non-small-cell lung cancer in patients on hemodialysis: clinical outcome and long-term results. World J Surg. 2005;29:1516–9. 16222451 10.1007/s00268-005-0047-4

[29] Yamauchi T Miyata H Sakaguchi T. Coronary artery bypass grafting in hemodialysis-dependent patients: analysis of Japan adult cardiovascular surgery database. Circ J. 2012;76:1115–20. 22333214 10.1253/circj.cj-11-1146

